# Mosquito swarms shear harden

**DOI:** 10.1140/epje/s10189-023-00379-3

**Published:** 2023-12-08

**Authors:** Andy M. Reynolds

**Affiliations:** Harpenden, AL5 2JQ Hertfordshire UK

## Abstract

Recently Cavagna et al. (Sci Rep 13(1): 8745, 2023) documented the swarming behaviors of laboratory-based *Anopheles gambiae* mosquitoes. Here key observations from this 3D-video tracking study are reproduced by a minimally structured (maximum entropy) stochastic trajectory model. The modelling shows that in contrast with midge swarms which are a form of collective behavior, unperturbed mosquito swarms are more like collections of individuals that independently circulate around a fixed location. The modelling predicts the observed response *Anopheles gambiae* mosquitoes in wild swarms to varying wind speeds (Butail et al. in J Med Entomol 50(3): 552–559, 2013). It is shown that this response can be attributed to shear hardening. This is because mosquitoes are found to be attracted to the centre of the swarm by an effective force that increases with increasing flight speed. Mosquitoes can therefore better resist the influence of environmental disturbances by increasing their flight speeds. This contrasts with other emergent mechanical-like properties of swarming which arise accidentally without a change in an individual’s behavior. The new results add to the growing realization that perturbations can drive swarms into more robust states.

**Supplementary Information:**

The online version contains supplementary material available at 10.1140/epje/s10189-023-00379-3.

Swarming is a natural behaviour in flying insects. The swarms typically show a high degree of spatial cohesion and are a form of collective animal behaviour; albeit one different from flocks and schools as they do not display ordered collective movements [1, 10, 13, 16]. Instead, each individual insect moves erratically and seemingly at random within the swarm. Their occurrence makes it clear that group order and morphology are not sufficient to accurately describe animal aggregations. Indeed, it is now recognized that the properties of a swarm, like inert material properties, cannot be determined by passive observation alone, instead one must interact with it, by for example applying controlled perturbations as pioneered by Nick Ouellette and his co-workers [11], [26, 27], [29, 30]. These experiments have revealed that swarms of *Chironomus riparius* midges have emergent properties such as an analogue of tensile strength that are reminiscent of thermodynamic and mechanical systems. Such properties have yet to be identified in other insect swarms. Here it is shown that the observed behaviours of wild swarms of male *Anopheles gambiae* mosquitoes [3] are consistent with the swarms undergoing ‘shear hardening’ in the presence of environmental disturbances, i.e., are consistent with the cohesive forces that bind individuals to the swarm centre strengthening as the environmental disturbances (wind shear etc.) increase. Swarms of females behave differently [4] and are not considered herein. The new results complement those of [19, 23] who hypothesised that the presence of a fluctuating environment drives the formation of transient, local order (synchronized subgroups) in mosquito swarms, and that this local order pushes the swarm into a new state that is robust to environmental perturbations. The results, old and new, suggest swarm state and structure may be tuneable with environmental noise as a control parameter.

In the next section I present a minimally structured (maximum entropy) model for the trajectories of swarming male mosquitoes. I show that this model is consistent with recent observations of swarming male *Anopheles gambiae* mosquitoes made under controlled, quiescent laboratory conditions [4]. In accordance with an analysis of pre-existing data [25] the model predicts that the swarming mosquitoes are attracted to the swarm centre by an effective speed-dependent force. I then show that the observed response *Anopheles gambiae* mosquitoes in wild swarms to increasing wind speeds [3] can be attributed to individuals *actively* increasing their flight speed and so becoming more tightly bound to the swarm center. Finally, I show how mosquito swarms may be poised at the cusps of disorder-order transitions (incoherent to coherent motion transitions).

Cavagna et al. [4] found that the vertical component of an individual’s velocity has a narrow distribution and that consequently motion mainly occurs on horizontal planes. Flying with gravity or upwards against gravity may be energetically costly. Here afterward attention is focused exclusively on the horizontal movements of male mosquitoes**.** Cavagna et al. [4] also reported that male mosquitoes move with near constant speed. The least biased (maximum entropy) choice for the distribution of individual’s speed is

1 $${p}_{s}={N}_{s}exp\left(-\frac{{\left(s-\overline{s }\right)}^{2}}{2{\sigma }_{s}^{2}}\right)$$

where ${N}_{s}$ is a normalization constant, $\overline{s }$ is a characteristic average speed of an individual and ${\sigma }_{s}$ is a measure of the variability in an individual’s speed. The least biased choice for the distribution of individual positions given only that the swarm is localized (on the origin) and coherent is a Gaussian with mean zero and variance ${\sigma }_{r}^{2}$. It is assumed that all individuals are characterised by the same values of $\overline{s }$, ${\sigma }_{s}$ and ${\sigma }_{r}$.

It follows from the analysis of Reynolds et al. [18] (see Supplementary Data 1) that the simplest radially symmetric two-dimensional minimally structured (maximum entropy) stochastic model for the joint evolution of the position, *x* and y, and velocity, *u* and v, of a mosquito within a swarm with Gaussian position statistics and homogeneous (spatially independent) Gaussian-like speed statistics (Eq. 1) is given by

2a $$du=-\frac{u}{Ts}\frac{{\sigma }^{2}}{{\sigma }_{s}^{2}}\left(s-\overline{s }\right)dt-\frac{{\mathrm{s}}^{2}}{{\sigma }_{r}^{2}}rsin\left(\phi -\theta \right)sin\left(\phi \right)dt+\sigma \sqrt{\frac{2}{T}d}{W}_{1}\left(t\right)$$

2b $$dv=-\frac{v}{Ts}\frac{{\sigma }^{2}}{{\sigma }_{s}^{2}}\left(s-\overline{s }\right)dt+\frac{{\mathrm{s}}^{2}}{{\sigma }_{r}^{2}}rsin\left(\phi -\theta \right)cos\left(\phi \right)dt+\sigma \sqrt{\frac{2}{T}d}{W}_{2}\left(t\right)$$

2c $$dx=udt$$

2d $$dy=vdt$$

where $x=rcos\left(\theta \right)$, $y=rsin\left(\theta \right)$, $u=s.cos\left(\phi \right)$, $v=s.sin\left(\phi \right)$, *r* is the distance from the swarm centre, *s* is the individuals speed, $\theta$ and $\phi$ are angular coordinates specifying the orientations of the mosquito’s position and velocity vectors, respectively, *T* is a velocity correlation timescale, $d{\varvec{W}}(t)$ is an incremental Wiener process with correlation property $\overline{d{W}_{i}\left(t\right)d{W}_{j}\left(t+\tau \right)}=\delta \left(\tau \right){\delta }_{ij}dt$ where the indices, *i,j* equal to 1 or 2, refer to Cartesian coordinates and where σ is of unit size and carries dimensions of speed.

The first term on the right-hand side of Eqs. (2a,b) is a memory term that causes velocity fluctuations to relax back to their mean value. The second terms on the right-hand sides of Eqs. (2a,b) are an effective force that binds individuals to the swarm center. The predicted position and speed dependency of this centrally attractive force is supported by empirical data (Supplementary Data 2). The noise term models a stochastic component of the internal forces that arise because of chance encounters with other individuals, and perhaps because of the inherent uncertainties in the detection of the ‘swarm marker’ (a visually prominent feature over which swarms form and are localized). Interactions between individuals are not modelled explicitly. Note that in the absence of noise $\left(\sigma =0\right)$ simulated individuals fly at constant speed, and the swarming pattern of a simulated individual consists of smooth elliptical loops, the foci of which gradually shift with respect to the centre of the swarm (the swarm marker) (Supplementary Data 3). The radial dependency of the ‘effective’ force is therefore indicative of centripetal like forces stemming perhaps from phototaxis. This contrasts with the case of swarming *Chironomus riparius* midges which are effectively confined within harmonic potential wells [10, 13], wells that are created by interspecific interactions [9, 20]. Because the mosquitoes are not confined within potential wells, the near constancy of speed implies near constancy of the observable energy (excluding expenditure of internal energy). Such constancy is unusual [15] and typically complicates model formation [24].

Equation 2 is the simplest of two possible minimally structured models. In this model the position and velocity vectors tend to be orthogonal which results in ring-like flight patterns. In the other more complicated model (presented in the Supplementary Data 4) position and velocity vectors tend to be parallel, resulting in flight patterns that pass back-and-forth through the swarm centre [18], (Supplementary Data 1).

The results of numerical simulations confirm that model predictions (model outputs) for the distributions of individual positions and speeds match the prescribed distributions (model inputs) (Fig. 1a, b) which characterize the observations of Cavagna et al. [4]. As observed [4] the distribution of a single component of velocity is predicted to be double peaked (Fig. 1c). Moreover, the model predicts ring-like or circular flight patterns of the kind reported on by Cavagna et al. [4] and earlier by Gibson [8] (Fig. 2). It has thereby been demonstrated, as claimed, that the model, Eq. 2, is consistent with distributions that characterise observations of individual positions and velocities, and it has been demonstrated that the model predicts correctly that individual mosquitoes have repetitive, pseudo-periodic movements.

**Fig. 1:**
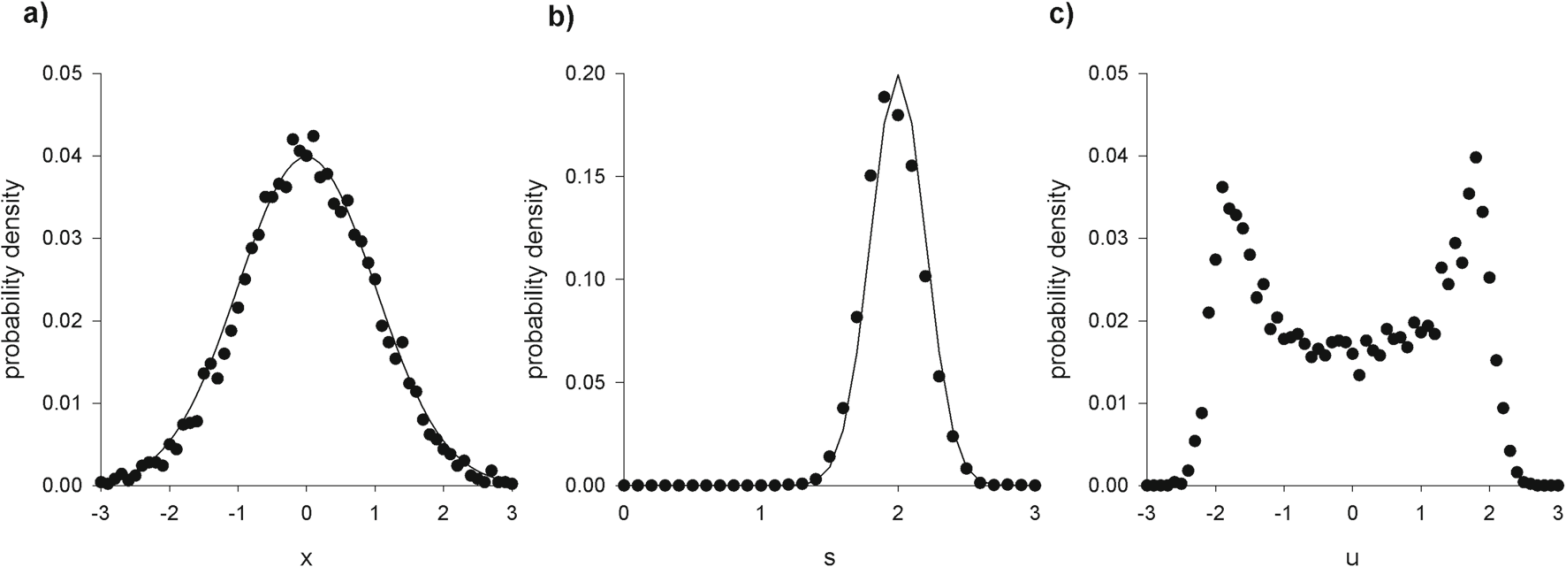
Model trajectories have the prescribed statistics. (**a**) and (**b**) The predicted positions and speeds of individual mosquitoes (•) match the prescribed distributions (solid lines) which encapsulate observations [4]. (**c**) In accordance with observations [4], the distribution of a single component of velocity is predicted to have two peaks. The apparent asymmetry is due to statistical noise and varies across simulations. Consequently, as observed [4] the two velocity components, u and v, lie within a narrow ring in u-v space. Predictions were obtained using the stochastic trajectory model, Eq. 2, with $\overline{s }=2, {\sigma }_{s}=\frac{1}{5},{\sigma }_{r}=1$ and $T=1$ a.u

**Fig. 2:**
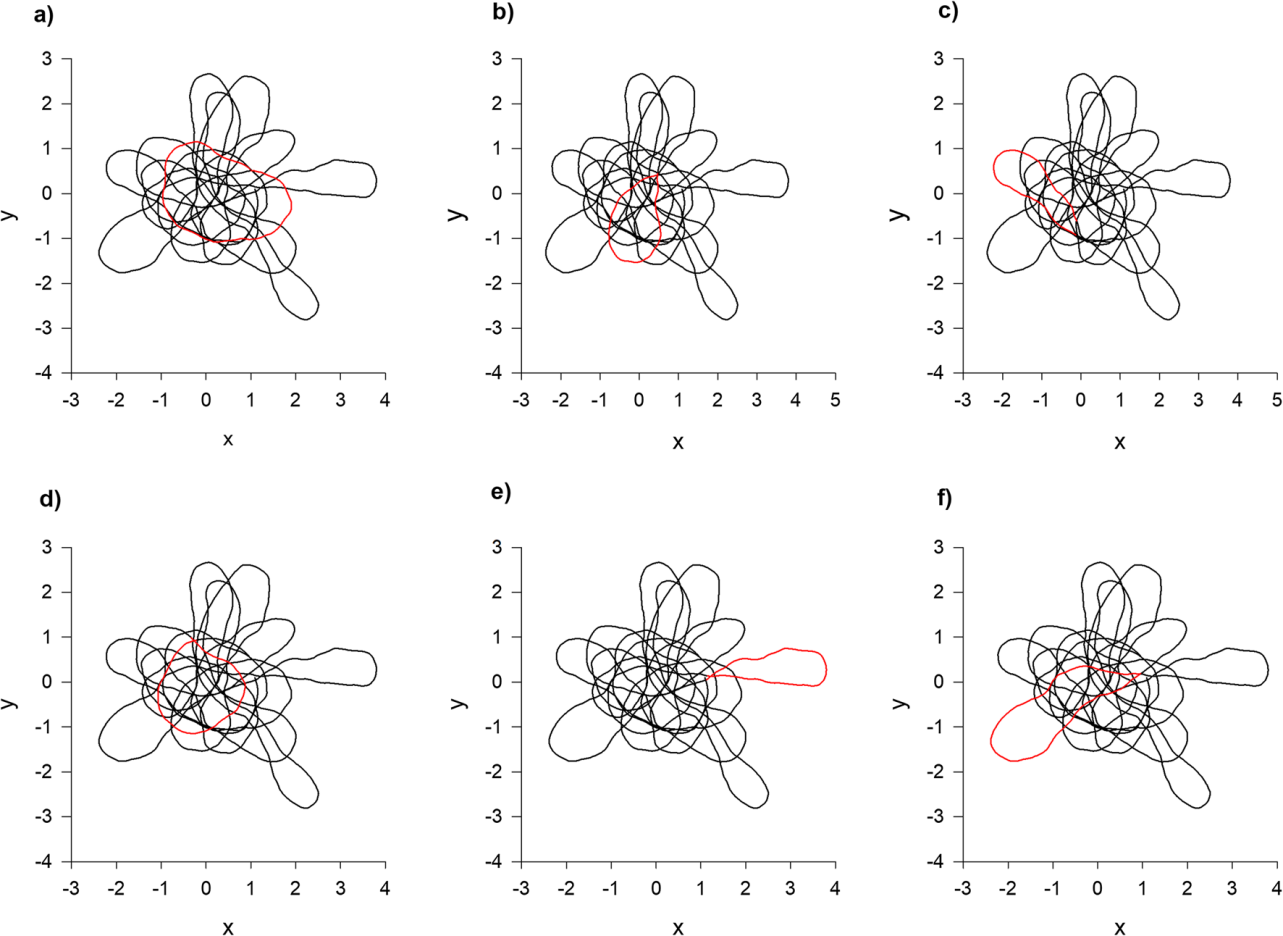
As observed [4], simulated individuals perform consecutive pseudo-circular motions. Here these are highlighted in red and are superimposed on the complete simulated trajectory for the same individual. Letters in the top corned of each sub-panel represent the temporal order of the ring-like movements. Predictions were obtained using the stochastic trajectory model, Eq. 2, with $\overline{s }=2, {\sigma }_{s}=\frac{1}{5},{\sigma }_{r}=1$ and $T=1$ a.u

Given this success, the model is now used to re-evaluate the observations of swarming mosquitoes made by Butail et al. [3] who utilized Okubo’s [13] classic model of swarming to interpret their findings. Okubo’s [13] model predicts that individuals tend to fly back-and-forth through the swarm centre rather than have ring-like flight patterns, and it predicts that individual velocities rather than speeds are Gaussian distributed. Butail et al. [3] reported that the horizontal movements of swarming mosquitoes are characterized by oscillatory velocity autocorrelation functions, $R\left(\tau \right)= \langle u\left(t\right)u\left(t+\tau \right)\rangle /\langle {u}^{2}\left(t\right)\rangle$ and that the frequency of oscillation increases with mean wind speed. The oscillatory velocity autocorrelation functions were taken to be indicative of underdamping. In the stochastic model, Eq. 2, oscillatory velocity autocorrelation functions result from the ring-like flight patterns (Fig. 2).

Butail et al. [3] fitted observed forms of the velocity autocorrelation function to the underdamped form predicted by Okubo’s [13] model,

3 $$R\left(\tau \right)={e}^{-{\omega }_{0}\xi \tau }\left(cos\left({\omega }_{1}\tau \right)-\frac{{\omega }_{0}\xi }{{\omega }_{1}}sin\left({\omega }_{1}\tau \right)\right)$$

where ${\omega }_{1}=\sqrt{1-{\xi }^{2}}$, ${\omega }_{0}$ is the natural frequency’ (a measure of the stiffness of the swarm) and $\xi$ is the ‘damping ratio’.

For generic second-order systems the damping ratio is independent of the natural frequency. Butail et al. [3] found that the damping ratio decreases monotonically as the natural frequency increases. Fitting model predictions from the stochastic model, Eq. 2, for the velocity autocorrelation function to Eq. 3 reveals that the observed relationship between damping ratio and natural frequency can be attributed to individuals *actively* responding to changes in the mean wind speed (Fig. 3), i.e., to individuals flying slowly when wind speeds are low and flying fast when wind speeds are high, even when flying upwind.

**Fig. 3:**
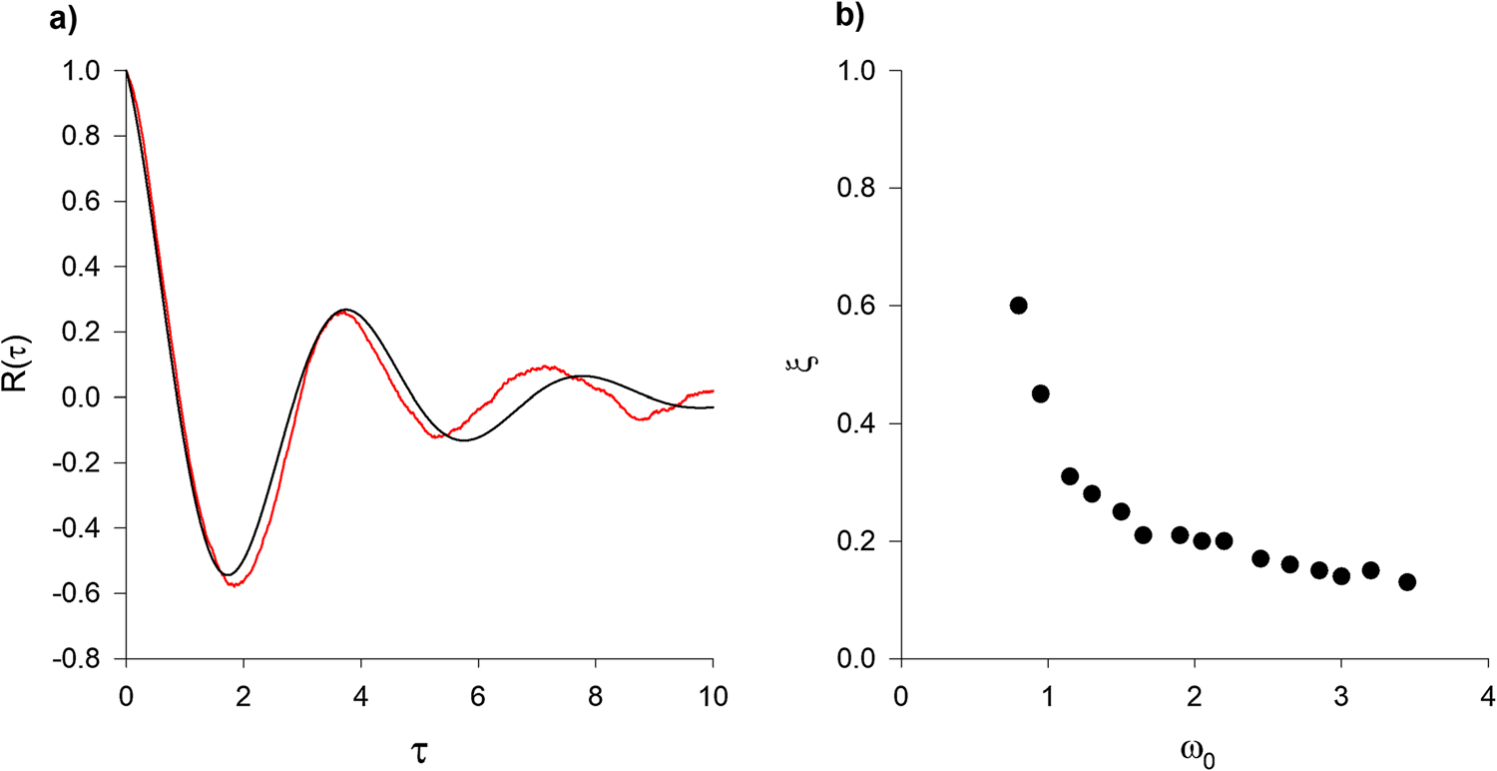
(**a**) As observed [4], velocity autocorrelations are oscillatory (black line). They can be accurately parameterized by Eq. 3 (red line). Predictions were obtained $\overline{s }=2, {\sigma }_{s}=\frac{1}{5},{\sigma }_{R}=1$ and $T=1$ a.u. (**b**) The predicted relationship between natural frequency, ${\omega }_{0}$, and the damping ratio, $\xi$, mirrors the observations of Butail et al. [3]. Predictions were obtained ${\sigma }_{s}=\frac{1}{5},{\sigma }_{r}=1$, $T=1$ a.u. and with mean speed $\overline{s }$ ranging between 1 and 4

Notice that the strength of the central attraction is predicted to increase as an individual’s speed increases (second terms on the right hand sides of Eqs. 2a, b). This together with the above re-analysis of the observations of Butail et al. [3] suggests that individual male mosquitoes can and do make the swarm more resistive to perturbations by increasing their mean speed when the wind speed increases. The swarm thereby shear hardens.

By way of contrast, midge swarms could in principle undergo shear hardening but in practise this ability may be unattainable because the strength of the central attraction has a weak dependency on mean speed [18]. Nonetheless, midge swarms have other seemingly advantageous, macroscopic mechanical-like properties that only become apparent when the swarms are perturbed [11], [29, 30]. These macroscopic mechanical-like properties although potentially advantageous arise spontaneously and do not necessitate those individuals actively responding to perturbations [21, 29].

The ring-like movements seen in the swarms of male mosquitoes [4] that stem from the effective speed-dependent force are a common feature of *Diptera* swarms [28]. They are also evident in Daphnia (commonly known as water fleas) [2], whirligig beetles [5] and in male honeybees (drones) [31]. These observations suggest that shear hardening is a common emergent property of insect swarms that helps to maintain cohesion and fault tolerance in the presence of environmental disturbances. Taken together various results old and new suggest that there are different pathways to swarm stabilization, some arising spontaneously without a change in an individual’s behavior [21, 22], [23], [29], some, such as shear hardening, requiring behavioral changes that may be the result of selection pressures for advantageous properties.

Because of their similitude with Daphnia, mosquito swarms may have the potential to form coherent vortices. Single Daphnia and Daphnia in dilute concentration circulate erratically and independently around light shafts [2]. Daphnia in high concentration circulate collectively in one direction, forming a vortex [7]. Their collective behaviour may be attributed to hydrodynamic coupling since the water inside the vortex turns in the same direction as the Daphnia [7]. Similarly, swarming mosquitoes could be coupled aerodynamically (Sanjay Sane -Privative Communication)—mosquitoes have long slender wings like birds which fly in formation and wherein all but the leading bird is positioned so that it can gain lift from the upwash generated by the wings of the preceding bird [17]. Collective motion in Daphnia and potentially in mosquitoes may also result from collision avoidance [14], [7], Mach and Schweitzer, Supplementary Data 5). But in practice a transition to a vortex state may be not realized by mosquitoes because flight formations are not sufficiently stable and/or because close encounters between individuals rarely occur [4]. Mosquito swarms may therefore be poised at the cusp of a disorder-order phase transition but always (setting aside mosquito ‘tornadoes’ [6] which may be the mosquito equivalent of dust devils) remaining on the disordered side because the vortex phase is not accessible to them. A very different form of swarming is predicted to occur when the effective Reynolds number of a swarm is sufficiently small (Supplementary Data 6).

### Supplementary Information

Below is the link to the electronic supplementary material.

Supplementary file1 (DOCX 894 kb)

## Data Availability

Data sharing not applicable to this article as no datasets were generated or analysed during the study.
