# Supplementary material for: Mosquito swarms shear harden
Source: Eur Phys J E Soft Matter. 2023 Dec 8;46(12):126. doi: 10.1140/epje/s10189-023-00379-3 (PMC10709253; doi:10.1140/epje/s10189-023-00379-3)
Supplement: Supplementary file 1 — Supplementary file1 (DOCX 894 kb) [file 10189_2023_379_MOESM1_ESM.docx]

**Supplementary Material**

**1. Derivation of the minimally structured (maximum entropy) stochastic model**

Here I devise a model that by construction is necessarily consistent with the observed kinematics of swarming mosquitoes, i.e., with distributions characterizing the observed positions and velocities of swarming mosquitoes.

I assume that the positions, ***x***, and velocities, ***u***, of individual mosquitoes can be described by the stochastic differential equations,

$\begin{matrix} du_{i}=a_{i}\left(u,x,t\right)dt+b_{ij}\left(u,x,t\right)dW_{j}\left(t\right) \\ dx_{i}=u_{i}dt \end{matrix}$ (S1)

where the subscripts denote Cartesian components and where $dW\left(t\right)$ is an incremental Wiener process with correlation property $\overline{dW_{i}\left(t\right)dW_{j}\left(t+\tau\right)}=\delta\left(\tau\right)\delta_{ij}dt$. I further assume that the magnitude of the driving noise,$b_{ij}\left(u,x,t\right)$, is a constant. Equation (S1) is effectively a first-order autoregressive stochastic process in which position and velocity are modelled as a joint Markovian process.

The deterministic term, $a\left(u,x,t\right)$, is here determined by the requirement that the statistical properties of the simulated positions and velocities be consistent with the experimental form of the mosquito’s density profile and velocity statistics. Mathematically, these consistency conditions require that $a_{i}\left(u,x,t\right)$ be a solution of the Fokker-Planck equation,

$\frac{\partial P}{\partial t}+u_{i}\frac{\partial P}{\partial x_{i}}=-\frac{\partial}{\partial u_{i}}\left(a_{i}P\right)+\frac{b^{2}}{2}\frac{\partial^{2}P}{\partial u_{i}^{2}}$ (S2)

where $P(u,x,t)$is the joint distribution of velocity and position [Thomson 1987]. For statistically stationary swarms (with $\partial P/\partial t=0$),

$a_{i}=\frac{b^{2}}{2}\frac{\partial\ln P}{\partial u_{i}}+\frac{\varphi_{i}}{P}$ (S3)

where

$\frac{\partial\varphi_{i}}{\partial u_{i}}=-u_{i}\frac{\partial P}{\partial x_{i}}$ (S4)

The first term on the right-hand side of Eqn. (S3) is a memory or relaxation term that drives velocities back towards their equilibrium values. The second term is effectively a conditional mean acceleration, $\left\langle A\left|u,x\right.\right\rangle$. The noise term in Eqn. S1, $bdW_{i}$, models a stochastic component of the internal forces that arise because of chance encounters with other individuals, and perhaps because of the inherent uncertainties in the detection of the ‘swarm marker’ (a visually prominent feature over which swarms form and are localized).

Laboratory swarms of mosquitoes are axisymmetric, and motion mainly occurs on horizontal planes [Cavagna et al. 2023]. In this case Eqn. (S4) is most naturally expressed in polar coordinates as

$\frac{\partial}{\partial s}\left(s\varphi_{s}\right)+\frac{\partial\varphi_{\phi}}{\partial\phi}=-s^{2}\cos\left(\phi-\theta\right)\frac{\partial P}{\partial r}$ (S5)

where $x_{1}=r\cos\theta$, $x_{2}=r\sin\theta$, $u_{1}=s\cos\phi$, $u_{2}=s\sin\phi$, , *r* is the radial distance from the swarm centre, *s* is the mosquito’s flight speed, , $\theta$ and $\phi$ are angular coordinates specifying the orientations of the mosquito’s position and velocity vectors respectively and $\hat{e}_{s}$and $\hat{e}_{\phi}$are unit vectors. For swarms with Gaussian density profiles centred on the origin and having root-mean-square width$\sigma_{r}$, $\frac{\partial P}{\partial r}=-\frac{r}{\sigma_{r}^{2}}$. A simple solution to Eqn. (S5) and the one used in the main text, Eqn 2, is given by

$$\varphi_{s}=0$$

$\frac{\varphi_{\phi}}{P}=\frac{s^{2}}{\sigma_{r}^{2}}\sin\left( \phi-\theta\right)$ (S6)

In this model, mean accelerations $s^{2}r/\sigma_{r}^{2}$are analogous to centripetal accelerations $s^{2}/r=\omega^{2}r$where $\omega$is the angular velocity. Simulated individuals tend to travel around the swarm marker.

Another simple model [see Supplementary Data 4] is

$\begin{matrix} \varphi_{\phi}=0 \\ \varphi_{s}=-\frac{\left(r-r_{c}\right)}{\sigma_{r}^{2}}\cos\left(\phi-\theta\right)\frac{1}{s}\int_{s}^{\infty} s^{2}Pds \end{matrix}$ (S6)

In these models, individuals tend to travel back-and-forth through the swarm centre.

**Reference not in main text**

Thomson, D.J. Criteria for the selection of stochastic models of particle trajectories in turbulent flows. *J. Fluid Mech.* **180**, 529-556 (1987).

**2. Empirical support for the predicted position- and speed-dependency of the centrally attractive force**

**
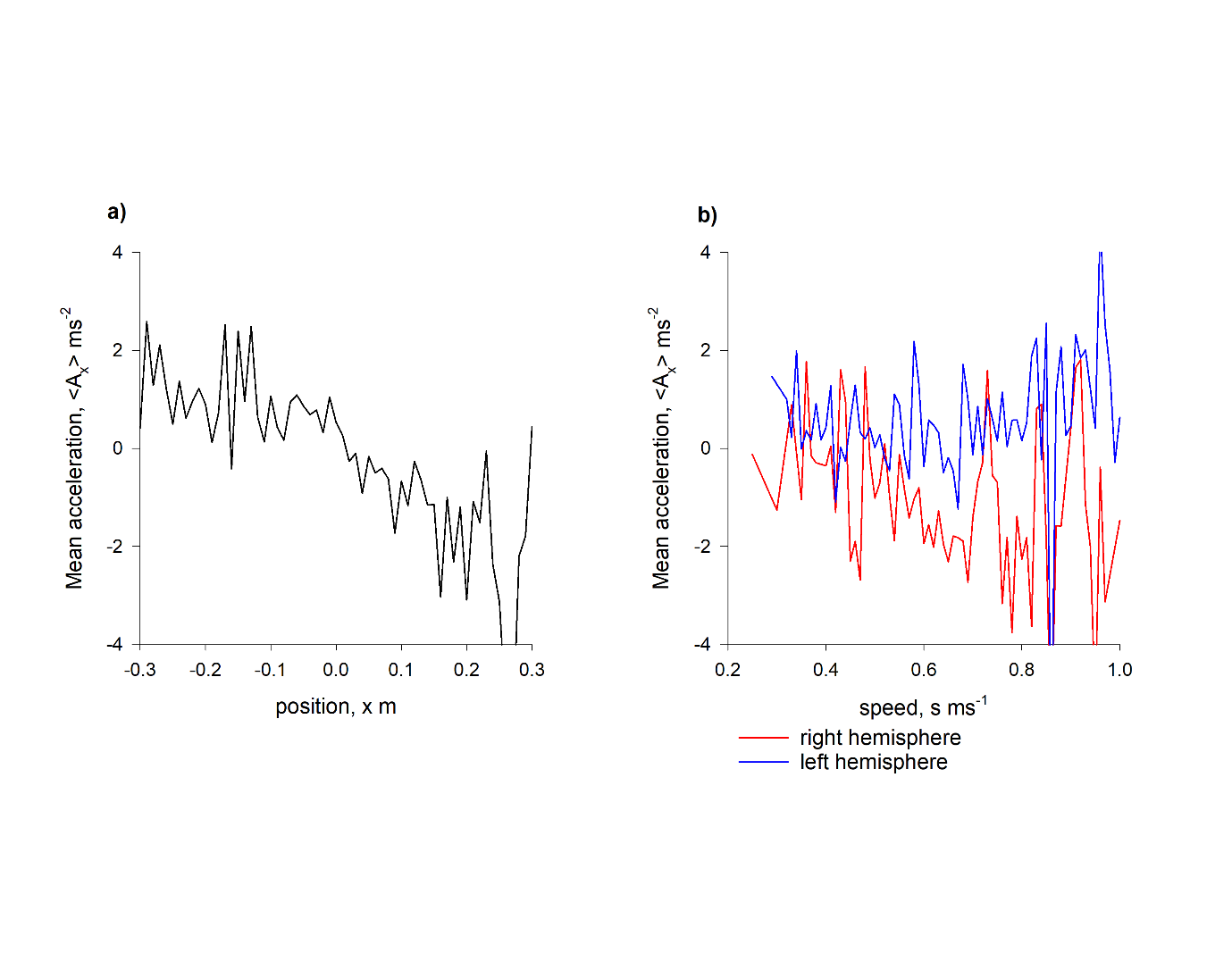
**

**Figure S1.** **Empirical data for the mean value of a single component of the mean acceleration, $\left\langle\boldsymbol{A}_{\boldsymbol{x}} \right\rangle$,** conditioned on the mosquito’s **a)** distance from the centre of the swarm, *x* and **b**) speed, *s*, computed from the largest data set, ‘male 8212010’, described in Shishika et al [2014]. The apparent asymmetry may be attributed to the relatively small size of the data set. The data set contains 27 trajectories each consisting of 270 positional fixes sampled regularly at 0.04 s intervals. As predicted the magnitude of the mean acceleration increases with increasing distance from the centre of the swarm and it increases with increasing flight speed. Shishika et al. [2014] did not report on mean accelerations.

**3. Swarming patterns in the absence of noise**

**

**

**Figure S2. In the absence of noise$\left( \boldsymbol{\sigma=0} \right)$ the swarming pattern of a simulated individual consists of smooth elliptical loops, the foci of which gradually shift with respect to the swarm maker**. The example pattern was obtained using the stochastic trajectory model, Eqn. 2, with $\bar{s}=2, \sigma_{s}=\frac{1}{5},\sigma_{r}=1$ and $T=1$ a.u.

**4. The Axial model**

In this section I present the alternative, more complicated minimally structured model and show that it predicts that swarms can shear harden and that they behave like auxetic materials.

It follows from the analysis of Reynolds et al. [2017] [see Supplementary Data 1] that the alternative (more complex) radially symmetric 2-dimensional minimally structured (maximum entropy) stochastic model for the joint evolution of the position, *x* and y, and velocity, *u* and v, of a mosquito within a swarm with Gaussian position statistics and Gaussian-like speed statistics (Eqn. 1) is given by

$du=-\frac{u}{Ts}\frac{\sigma^{2}}{\sigma_{s}^{2}}\left( s-\bar{s} \right)dt-\frac{\sigma_{s}^{2}}{\sigma_{r}^{2}}r\left( 1+\sqrt{\frac{\pi}{2}}\frac{\bar{s}}{\sigma_{s}} \frac{erfc\left( \frac{s-\bar{s}}{\sqrt{2}\sigma_{s}} \right)}{exp\left( -\frac{\left( s-\bar{s} \right)^{2}}{2\sigma_{s}^{2}} \right)} \right)cos\left( \phi-\theta\right)cos\left( \phi\right)dt+\sigma\sqrt{\frac{2}{T}d}W_{1}\left( t \right)$

$dv=-\frac{v}{Ts}\frac{\sigma^{2}}{\sigma_{s}^{2}}\left( s-\bar{s} \right)dt-\frac{\sigma_{s}^{2}}{\sigma_{r}^{2}}r\left( 1+\sqrt{\frac{\pi}{2}}\frac{\bar{s}}{\sigma_{s}} \frac{erfc\left( \frac{s-\bar{s}}{\sqrt{2}\sigma_{s}} \right)}{exp\left( -\frac{\left( s-\bar{s} \right)^{2}}{2\sigma_{s}^{2}} \right)} \right)cos\left( \phi-\theta\right)sin\left( \phi\right)dt+\sigma\sqrt{\frac{2}{T}d}W_{2}\left( t \right)$

$$dx=udt$$

(S8)

$dy=vdt$

where $x=rcos\left( \theta\right)$, $y=rsin\left( \theta\right)$, $u=scos\left( \phi\right)$, $v=ssin\left( \phi\right)$, *r* is the distance from the swarm centre, *s* is the individual’s speed, $\theta$ and $\phi$ are angular coordinates specifying the orientations of the mosquito’s position and velocity vectors respectively, *T* is a velocity correlation timescale, $d\boldsymbol{W}(t)$ is an incremental Wiener process with correlation property $\overline{dW_{i}\left( t \right)dW_{j}\left( t+\tau\right)}=\delta\left( \tau\right)\delta_{ij}dt$ and where σ is of unit size and carries dimensions of speed.

The results of numerical simulations confirm that model predictions for the distributions of individual positions and speeds match the prescribed distributions (Fig. S3a,b) and moreover that the distribution of a single component of velocity is, as observed [Cavagna et al. 2023] double peaked (Fig. S3c), indicating that the two velocity components, *u* and *v*, are predicted, as observed [Cavagna et al. 2023], to lie within a narrow ring in *u-v* space, which in turn is indicative of individuals moving with near constant speed.

For speeds close to the mean speed the magnitude of the central attraction$, \frac{\sigma_{s}^{2}}{\sigma_{r}^{2}}r\left( 1+\sqrt{\frac{\pi}{2}}\frac{\bar{s}}{\sigma_{s}} \frac{erfc\left( \frac{s-\bar{s}}{\sqrt{2}\sigma_{s}} \right)}{exp\left( -\frac{\left( s-\bar{s} \right)^{2}}{2\sigma_{s}^{2}} \right)} \right)$, is, to good approximation, given by $-\frac{\sigma_{s}^{2}}{\sigma_{r}^{2}}r\left( 1+\sqrt{\frac{\pi}{2}}\frac{\bar{s}}{\sigma_{s}} \right)$, i.e., the magnitude of the central attraction increases as the mean speed increases. The model therefore predicts that swarms have the potential to shear harden (see main text).

The results of numerical simulations show that this model, in contrast with the simpler model, Eqn. 2, predicts that swarms behave like auxetic (negative Poisson ratio) materials, i.e., when they are stretched (by momentarily shifting the positions of individuals from their equilibrium positions, $x\to2x)$, they become thicker in the direction perpendicular to the applied force (Fig. S4). Midge swarms are also predicted to be auxetic via a different pathway involving multiplicative noise [Reynolds 2021].

It remains to be seen whether this more complicated minimal model is realized in practice by some species of insect. In such cases individuals would like midges [Okubo 1986, Kelley and Ouellette 2013] be effectively confined by potential wells, albeit in 2- rather than 3-dimensions.





**Figure S3. Model trajectories have prescribed statistics. a and b)** The predicted positions and speeds of individual mosquitoes (•) match the prescribed distributions (solid lines) which encapsulate observations [Cavagna et al. 2023]. **c)** In accordance with observations [Cavagna et al. 2023] the distribution of a single component of velocity is predicted to have two peaks. Predictions were obtained using the stochastic trajectory model, Eqn. S8, with $\bar{s}=1, \sigma_{s}=\frac{1}{5},\sigma_{r}=1$ and $T=1$ a.u.

**

**

**Figure S4.** **Swarms are predicted to be auxetic.** After being stretched by a factor 2 in the x-direction at time t=0, **a)** the size of the simulated swarm in the x-direction, $\sigma_{x}$, relaxes monotonically back to equilibrium whilst **b)** the size of the swarm in the orthogonal direction increases for a time. Swarms are therefore predicted to behave like auxetic materials. Predictions were obtained using the stochastic trajectory model, Eqn. S8, with $\bar{s}=1, \sigma_{s}=\frac{1}{5},\sigma_{r}=1$ and $T=1$ a.u. Similar predictions are obtained if instead of the sudden expansion at *t=0*, the swarm gradually is pulled apart in the x-direction by the application of fictitious forces acting over an extended time interval.

**5. Mutual repulsion leads to self-organized collective motion - coherent vortices**

The results of numerical simulations indicate that repulsion allows for breaking of the symmetry between clockwise and anticlockwise motion, leading to the formation of vortices, an archetypical form of self-organized collective motion (Fig. S5a,b). Predictions were obtained by adding a repulsion term, $-\sum_{j=1}^{N} \frac{\boldsymbol{r}_{ij}}{r_{ij}^{2}+\varepsilon}dt$ to the right-hand side of the stochastic trajectory simulation model, Eqn. 2, where $r_{ij}$ is the distance between individuals ‘*i*’ and ‘j’ and where $\varepsilon=0.01$ is introduced to prevent any potential singularities. Here it is tactfully assumed that repulsion does not change flight dynamics captured by Eqn. 2. The results of numerical simulations reveal that symmetry breaking does not arise spontaneously in the presence of this kind of repulsion. Instead, symmetry breaking occurs if most individuals are momentarily set in rotation around the swarm centre (as might happen following a disturbance) and is most prevalent at high speeds (Fig S5c), i.e., following a perturbation that induces shear hardening (main text). Put differently, once established global rotations persist indefinitely in the presence of repulsions when individuals are flying sufficiently fast, but not otherwise. At low speeds, repulsion together with the presence of a fluctuating environment drives the formation of transient, local order (synchronized subgroups) of the kind seen in wild mosquito swarms [Reynolds 2023].

Finally, note that rotational symmetry is predicted to be broken spontaneously if each individual term $-\frac{\boldsymbol{r}_{ij}}{r_{ij}^{2}+\varepsilon}dt$ in the repulsion overall term $-\sum_{j=1}^{N} \frac{\boldsymbol{r}_{ij}}{r_{ij}^{2}+\varepsilon}dt$ is only applied if individual ‘j’ is moving togethers individual ‘i’ (results not shown). The revised term mimics a behavioural response akin to collision avoidance rather than a physical repulsion. As with simple repulsion, rotational symmetry is most prevalent at high speeds.

This study complements that of D'Orsogna et al. [2006], Chuang et al. [2007], Kolokolnikov et al. [2011], Bertozzi et al. [2015] who showed that short range repulsion together with long range pairwise attraction can result in ring shaped swarms. Note, however, that it is likely that unperturbed swarms of mosquitoes, like unperturbed swarms of midges [Puckett et al. 2014, van der Vaart et al. 2020] display short range repulsion but not exhibit long-range pairwise attraction.

**References not in the main text**

Bertozzi, A.L. et al. Ring patterns and their bifurcations in a nonlocal model of biological swarms. *Comm. Math. Sci.* **12**, 995-985 (2015).

Chuang Y. et al. State transitions and the continuum limit for a 2D interacting, self-propelled particle system. *Physica D* **232**, 33-47 (2007).

D'Orsogna, M.R. et al. Self-Propelled Particles with Soft-Core Interactions: Patterns, Stability, and Collapse.  *Phys. Rev. Lett.* **96**, 104302 (2006).

Kolokolnikov, T. et al. Stability of ring patterns arising from two-dimensional particle interactions, *Phys. Rev. E* **84**, 015203 (2011).

**

**

**Figure S5. Repulsion allows for breaking of the symmetry between clockwise and anticlockwise motion, leading to the formation of vortex, an archetypical form self-organized collective motion. a)** An example of a simulated trajectory of the individual in the vortex state. **b)** The distribution of angular momentum of all simulated individuals in the vortex state – showing a propensity for anticlockwise motion **c)** The percentage of individuals with anticlockwise motion as a function of an individual’s mean speed – showing that symmetry breaking occurs most readily at high speed. Predictions were obtained by adding a repulsion term, $-\sum_{j=1}^{N} \frac{\boldsymbol{r}_{ij}}{r_{ij}^{2}+\varepsilon}dt$ to the right-hand side of the stochastic trajectory simulation model, Eqn. 2, where $r_{ij}$ is the distance between individuals ‘*i*’ and ‘*j*’ and where $\varepsilon=0.01$ is introduced to prevent potential singularities. Results in panels a) and b) are shown for $\bar{s}=2, \sigma_{s}=\frac{1}{5},\sigma_{r}=1$ and $T=1$ a.u. Results in panels are shown for with $\frac{1}{2}\leq\bar{s}\leq10, \sigma_{s}=\frac{1}{5},\sigma_{r}=1$ and $T=1$ a.u. Initially all individuals right (left) of the swarm centre were moving upwards (upwards).

**6. Reynolds number effects**

In this section I show that insect swarms are predicted. as anticipated by Reynolds (2023b), to undergo an ordered to disordered transition as their effective Reynolds number increases.

In the above models the positions and velocities of a swarming mosquito are modelled jointly as a Markovian process, i.e., as a first-order autoregressive process. Here attention is focused on a higher-order variant of these models in which the positions, velocities and accelerations of a swarming insect are collectively Markovian, i.e., are modelled as second-order autoregressive processes. Physically, such modelling corresponds to the inclusion of a time scale, *T*, representative of the largest scales of motion, at first order, and the addition of a time scale, $t_{s}$, representative of the smallest scale of motion, at second order. An effective Reynolds number, defined by the ratio of these time scales, $R=\left( T/t_{s} \right)^{2}$ , therefore appears as a parameter at second order [Sawford 1991]. Strong evidence for second-order effects consistent with model expectations can be found in Reynolds [2020] who analysed laboratory data for swarming midges [Sinhuber et al. 2019]. Reynolds [2020] found intriguingly similarities between the acceleration statistics of swarming midges and the acceleration statistics of tracer particles in high Reynolds number turbulence.

It follows from the analysis of Reynolds et al. [2017,2020] that the simplest radially symmetric 2-dimensional minimally structured (maximum entropy) stochastic model for the joint evolution of the position, *x* and y, velocity, *u* and v, and acceleration *A_x_* and *A_y_* of a mosquito within a swarm with Gaussian position statistics, homogeneous (spatially independent) Gaussian-like speed statistics (Eqn. 1) and Gaussian acceleration statistics is given by

$d\acute{A}_{x}=-\frac{\acute{A}_{x}}{\hat{T}}dt-{sin\left( \xi\right) \left| \acute{\boldsymbol{A}} \right|}^{2}\frac{\left( s-\bar{s} \right)}{\sigma_{s}^{2}}sin\left( \xi-\phi\right)dt+\sigma_{A}\sqrt{\frac{2}{\hat{T}}d}W_{1}\left( t \right)$ (S9)

$d\acute{A}_{y}=-\frac{\acute{A}_{y}}{\hat{T}}dt+cos\left( \xi\right)\left| \acute{\boldsymbol{A}} \right|^{2}\frac{\left( s-\bar{s} \right)}{\sigma_{s}^{2}}sin\left( \xi-\phi\right)dt+\sigma_{A}\sqrt{\frac{2}{\hat{T}}d}W_{2}\left( t \right)$

$dx=udt$

$dy=vdt$

$$du=\left( \bar{A}_{x}+\acute{A}_{x} \right)dt$$

$$dv=\left( \bar{A}_{y}+\acute{A}_{y} \right)dt$$

$$\bar{A}_{x}=-sin\left( \phi\right)\frac{sr}{\sigma_{r}^{2}}sin\left( \phi-\theta\right)$$

$$\bar{A}_{y}=cos\left( \phi\right)\frac{sr}{\sigma_{r}^{2}}sin\left( \phi-\theta\right)$$

where $x=rcos\left( \theta\right)$, $y=rsin\left( \theta\right)$, $u=scos\left( \phi\right)$, $v=ssin\left( \phi\right)$, $\acute{A}_{x}=\left| \acute{\boldsymbol{A}} \right|cos\left( \xi\right)$, $\acute{A}_{y}=\left| \acute{\boldsymbol{A}} \right|sin\left( \xi\right)$ *r* is the distance from the swarm centre, *s* is the individuals speed, $\theta$ and $\phi$ are angular coordinates specifying the orientations of the mosquito’s position and velocity vectors respectively, $\hat{T}$is an acceleration correlation timescale ($\hat{T}^{-1}=T^{-1}+t_{s}^{-1} [$Sawford 1991]), $d\boldsymbol{W}(t)$ is an incremental Wiener process with correlation property $\overline{dW_{i}\left( t \right)dW_{j}\left( t+\tau\right)}=\delta\left( \tau\right)\delta_{ij}dt$. In the simplest second-order model, the acceleration and velocity variances are related by $\sigma_{A}^{2}=\frac{\sigma_{u}^{2}}{Tt_{s}}$ [Sawford 1991]. Here, following Sawford [1991], the acceleration variance, which appears as a free parameter in the above model, is taken to given by $\sigma_{A}^{2}=\frac{\bar{S}^{2}}{Tt_{s}}$ . As a consequence, fluctuations in the strength of central attraction decrease as *R* decreases, and vanish when *R=0*.

The results of numerical simulations confirm that model predictions for the distributions of individual positions, speeds and accelerations match the prescribed distributions (Fig. S6). In this model, as in its first-order counterpart Eqn. 2, the position and velocity vectors tend to be orthogonal. The velocity and fluctuating acceleration vectors also tend to be orthogonal. Therefore, the position and the fluctuating acceleration vectors tend to be parallel, i.e., the mean acceleration and fluctuating acceleration vectors tend to be parallel. In accordance with observations [Cavagna et al. 2023], the model, Eqn. S9, predicts that flight patterns are ring-like (Fig.S7). At low Reynolds numbers, individual trajectories are seen to be nearly periodic whilst at high Reynolds numbers they are less structured. The model therefore predicts, as anticipated by Reynolds [2023], that swarms undergo an ordered to disordered transition as the swarm’s effective Reynolds number increases. The nature of the transition is quantified in Fig. S8a which shows that the power-spectrum of positions is peaked at low Reynolds numbers but not at high Reynolds numbers. The transition is also evident in trajectory curvature statistics (Fig. S8b). Note that here ‘order’ does not imply coherent motion as individual movements are not correlated and so the transition is distinctly different from the order to disorder (coherent to incoherent motion) envisaged by Cavagna et al. [2023], albeit in the context of midges rather than mosquitoes. Coherent motion in insect swarms of has not been reported on.

It is noteworthy that the average jerks (second terms on the right-hand side of the equations for $d\acute{\boldsymbol{A}}$) tend to be aligned with velocity, i.e., tend to be aligned along the body of the insect. Their potentially destabilizing influence on flight control is thereby minimized. This is also true of swarming midges [Reynolds 2020]. Note also that when *R=0*, i.e., when $\sigma_{A}=0$, the stochastic noise terms in Eqn. S9 vanishes, simulated trajectories become deterministic, conserve kinetic energy and maybe chaotic (Fig. S9). This form of chaos is distinctly different from the chaos found recently in the harmonically confined Vicsek model – a model of insect swarming advocated by González-Albaladejo et al. [2023].

**References not in main text**

Cavagna et al. Natural swarms in 3.99 dimensions. *Nat. Phys*. **19**, 1043-1049 (2023).

##### González-Albaladejo, R., Carpio, A. & Bonilla, L.L. Scale free chaos in the confined Vicsek flocking model*. Phys. Rev. E.* **107**, 014209, 2023.

Reynolds, A.M. Insect swarms can be bound together by repulsive forces. *Eur. Phys. J. E*. **43**, 39, 2020.

Sawford, B.L. Reynolds number effects in Lagrangian stochastic models of turbulent dispersion*. Phys. Fluids* A **3** 1577-1586, 1991.

Sinhuber, M., van der Vaart, K., Ni, R., Puckett, J.G., Kelley, D.H. & Ouellette, N.T. Three-dimensional time-resolved trajectories from laboratory insect swarms. *Sci. Data* **6**, 190036 (2019).





**Figure S6. Model trajectories have prescribed statistics. a), b) and c)** The predicted positions, speeds, and fluctuating components of accelerations of individual mosquitoes (•) match the prescribed distributions (solid lines) which encapsulate observations [Cavagna et al. 2023]. Predictions were obtained using the stochastic trajectory model, Eqn. S9, with $\bar{s}=2, \sigma_{s}=\frac{1}{5},\sigma_{r}=1,$ $\sigma_{A}^{2}=2$ and $\hat{T}=1$ a.u.





**Figure S7. Examples of simulated trajectories for low and high effective Reynolds numbers.** Predictions were obtained using the stochastic trajectory model, Eqn. S9, with $\bar{s}=2, \sigma_{s}=\frac{1}{5},\sigma_{r}=1,$ $T=1$ , ${\left( a \right) t}_{s}=1/50$ and (b) $t_{s}=1$ a.u., corresponding to Reynolds numbers $R\boldsymbol{=}{(\frac{1}{50})}^{2}$ and $R=1$.

**
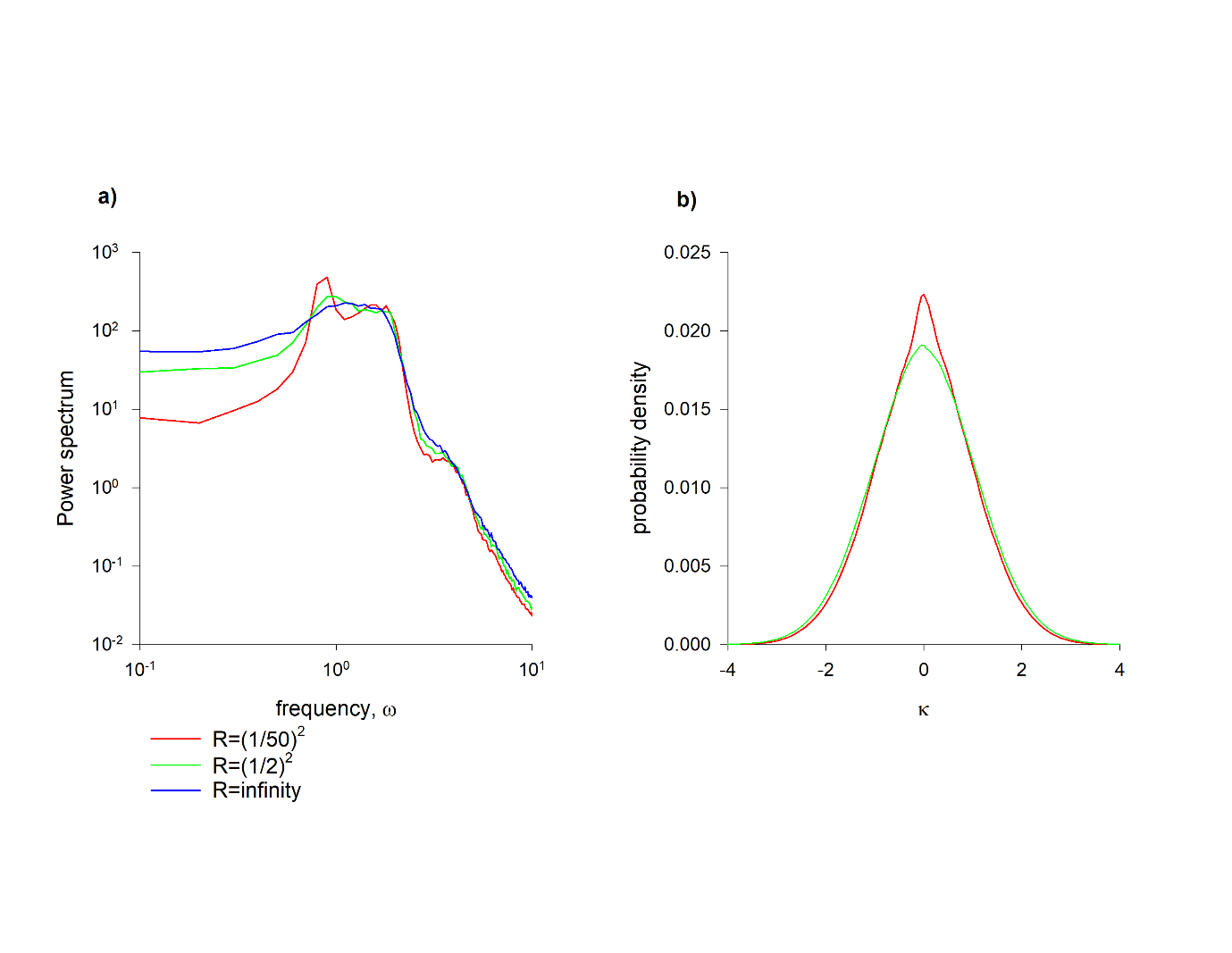
**

**Figure S8. Two indicators of an order to disorder transition a)** The power spectrum is predicted to be peaked at low Reynolds numbers but not at high Reynolds numbers. This is indicative of an order to disorder transition. **b)** Distribution of the reciprocal of the radius of curvature, $\kappa=\left( uA_{y}-vA_{x} \right)/\left( u^{2}+v^{2} \right)^{3/2}$. The distribution is more strongly peaked at low Reynolds. This is indicative of straighter trajectories. Predictions for finite Reynolds numbers (finite acceleration variances) were obtained using the stochastic trajectory model, Eqn. S9, with $\bar{s}=2, \sigma_{s}=\frac{1}{5},\sigma_{r}=1$, $T=1$, $t_{s}=\frac{1}{50}$ (red) and $t_{s}=\frac{1}{2}$ (green) a.u. Predictions for infinite Reynolds number (blue) were obtained with the stochastic trajectory model, Eqn. 2, with $\bar{s}=2, \sigma_{s}=\frac{1}{5},\sigma_{r}=1$and $T=1$ a.u.

**

**

**Figure S9. Simulated trajectories may be chaotic when R=0.** The separation between individual mosquitoes, that are initially close together in phase space, increases in a near exponential way at short times (•). Exponential growth of the separation in phase space is indicative of chaos. Such growth cannot continue indefinitely because the volume of the phase space is bounded. The solid-line is a linear regression on the log-linear scales and is indicative of exponential growth. *R^2^=0.98.* Predictions were obtained using the stochastic trajectory model, Eqn. S9, with $\bar{s}=2, \sigma_{s}=\frac{1}{5},\sigma_{r}=1,$ $\sigma_{A}^{2}=2$ and $T=1$ a.u. Predictions shown correspond to the largest Lyapunov exponent.
